# Supplementary material for: Are carbon emissions trading and green financial instruments synergistic? -Comprehensive quantitative research based on content analysis
Source: PLoS One. 2024 Mar 7;19(3):e0298601. doi: 10.1371/journal.pone.0298601 (PMC10919601; doi:10.1371/journal.pone.0298601)
Supplement: S1 Data — (ZIP) [file pone.0298601.s001.zip › Data/地级市Z_F工作报告.zip!/╣π╢1⁄2/╓Θ║ú╩╨/└┤╫╘╣1⁄2╓┌║┼í╛┬φ┐╦╩2╛▌═°í┐_2007.docx]

各位代表：

　　现在，我代表市人民政府向大会作政府工作报告，请予审议，并请各位政协委员和其他列席人员提出意见。

　　一、过去三年工作回顾

　　过去三年，是我市经济社会发展取得显著成绩的三年。市政府在省委、省政府和市委的正确领导下，在市人大、市政协监督支持下，以邓小平理论和“三个代表”重要思想为指导，认真落实科学发展观，努力构建和谐社会，切实履行政府职能，开创了珠海现代化建设新局面，为今后五年发展打下了坚实的基础。

　　（一）经济保持较快发展，经济质量逐步提高。

　　经济增长速度加快。实施“工业强市”战略成效显著，三年间工业总产值实现了从1000亿元到2000亿元的跨越。第三产业稳步发展，旅游、房地产、汽车、通讯、文化、教育成为消费热点。农业和农村经济结构加快调整，培育了一批农业园区、农业龙头企业和特色农产品。2006年，实现全市本地生产总值749.6亿元，人均生产总值达5.2万元，三年年均分别增长14.6％和11.6％。全口径财政收入达195.2亿元，其中地方财政一般预算收入达到60.3亿元，年均增长21％。社会消费品零售总额255.5亿元，年均增长15.1％。固定资产投资总额255亿元，三年累计达656.2亿元，年均增长21.8％。进出口总额达328.2亿美元，其中出口148亿美元，出口增幅在珠三角城市中排名第一。去年末金融机构本外币存款余额达1148.7亿元，年均增长15.4％，珠海入选“中国十佳金融生态城市”。

　　经济质量明显提升。经济结构调整取得成效，工业适度重型化、高级化趋势明显，2006年全市重工业和高新技术产品产值分别占工业总产值的60.9％和38.4％。经省认定的高新技术企业增加到185家，省级以上工程技术研发中心和企业技术中心增加到27家，31项科技成果获国家和省科技进步奖。全市专利申请量、名牌产品及驰名商标数量均实现三年翻番，连续三年被评为“全国科技进步先进市”，格力空调荣获“中国世界名牌产品”。节能降耗卓有成效，单位生产总值能耗比全省平均水平低16.5％，列全省第三位。区域经济发展不平衡得到改善，香洲区发展势头良好，金湾、斗门两区占全市经济比重明显增加。

　　（二）基础设施建设取得重要进展，环境保护成绩突出。

　　城市基础设施日益完善。广珠城际轨道建设进展顺利，广珠铁路将于近期复工。港珠澳大桥项目前期工作有新进展。高栏港区两个5万吨级集装箱码头、公用石化码头等工程进展顺利。珠海机场和香港机场成功合作。西部沿海高速公路珠海段竣工通车，江珠、太澳高速公路珠海段即将通车。市内骨干路网建设力度加大，新建和改造机场北路、105国道上冲段等一批主干道，三年累计改造公路230多公里。珠海电厂3、4号机组和深能洪湾电厂投产，新增发电能力156万千瓦。横琴中海油天然气接收站投入运营。一批重点水闸和海堤险段得到加固，城乡水利防灾减灾能力大大提高。日调水100万吨的咸期应急供水工程竣工通水，保障了珠澳两地供水。

　　“生态珠海”建设成效明显。全国环境优美镇和生态示范镇、村创建工作初见成效。一批城中旧村改造成为新社区，一批烂尾楼工程复工建成。加强城市环境综合治理，加大治污保洁和江河水整治力度，市区污水处理率达到63％以上。海洋生态建设取得新进展，红树林湿地面积扩大。连续获得全省环保责任考核总分第一名、全省珠江综合整治考核第一名，在城市环境综合整治定量考核中名列全国前茅，通过“国家环保模范城市”复查，创建“全国生态示范市”工作进展顺利。

　　（三）改革迈出新步伐，开放取得新成果。

　　经济体制改革逐步深化。积极推进政府管理创新，完成新一轮机构改革和行政审批制度改革，清理和调整行政许可事项348项。实施新的城市管理体制，理顺了高新区、高栏港经济区管理体制。设立市、区行政服务中心，集中、公开、高效的办事模式受到社会广泛好评。进一步改革和完善国有资产管理体制，国有资本战略布局调整和结构优化取得成效，国有资产保值增值率逐年提高。民营经济呈现快速发展态势，民营企业对增加就业和财税收入等贡献突出。清理市直党政机关和事业单位资产达50多亿元。城市管道燃气等市政公用事业特许经营改革顺利推进。组建城市资产经营公司，加强城市公共资产资本化运营。金融改革取得新进展，市商业银行、农信社体制改革顺利进行，完成4家上市公司股权分置改革，新增6家企业成功上市。

　　对外开放水平不断提高。利用外资和对外贸易规模显著扩大，三年累计实际利用外商直接投资19.6亿美元，年均增长29.3％。珠澳跨境工业区珠海园区正式启用，横琴岛开发建设总体规划通过省政府评审。横琴口岸新联检楼落成，拱北口岸客车“一站式”验放系统和港澳旅客自助查验系统投入运行。引进内资成效显著，一批内地知名企业落户珠海。成功举办第五、第六届中国航展，高水平承办国际跳水大奖赛、国际汽联锦标赛等重大赛事，提升了珠海的国际知名度。

　　（四）公共资源管理加强，清还欠款工作成绩显著。

　　统筹公共资源水平提高。理顺规划管理体制，强化了规划的龙头作用。完成国土管理体制改革，健全土地管理决策机制和土地储备制度，土地储备总量达59.9平方千米，三年累计回收城市土地开发投资82.9亿元。全面实行部门预算，加强和规范非税收入管理。改革公务用车制度，有效节约行政成本。

　　清欠工作取得积极成果。全面兑付1988年至2004年拖欠的2.5亿元征地补偿款，清理建筑领域拖欠农民工工资超过1亿元，累计偿还政府工程欠款26.3亿元，维护了政府诚信形象。珠光公司债务重组进入实施阶段，珠海机场债务重组工作基本完成，为企业发展创造了良好条件。

　　（五）社会各项事业稳步发展，人民生活水平进一步提高。

　　教育、卫生、文化、体育事业全面发展。提前一年实现创建广东省“教育强市”目标，撤并改造25所农村薄弱学校，5.5万多名农村学生享受免费义务教育，创建4所国家级示范性普通高中，组建珠海城市职业技术学院。高等教育快速发展，全日制在校生人数达6.3万人，规模居全省第二。市人民医院、市妇幼保健院、市第二人民医院进行改扩建，市中心血站投入使用。在全国率先建设“三位一体”、“四网合一”的农村卫生服务中心。全面推行新型农村合作医疗制度，覆盖面达88％。食品药品安全监管工作得到加强。市图书馆和青少年妇女儿童活动中心投入使用，古元美术馆建设顺利，“陈芳家宅”、“宝镜湾遗址”列入全国重点文物保护单位，南方文化产业园发展势头良好，完成10万户有线数字电视整体转换，文化事业和文化产业全面发展。兴建了一批城乡文化体育设施，全民健身运动广泛开展，在省运会取得好成绩。精神文明建设取得丰硕成果，再次荣获“省文明城市”称号。

　　改善民生落到实处。大力实施就业培训工程，帮助5278名就业困难人员实现就业，转移农村富余劳动力就业累计33046人次，城镇登记失业率控制在3％以内。社会保障体系逐步健全，覆盖面明显扩大。推行农村养老保险，建立未成年人医疗保险制度，2006年全市养老、失业、工伤、医疗、生育等五项险种参保达254.3万人次。加大医疗救助和扶助贫困残疾人的力度，及时足额发放低保金、救济金和优抚金。在全省率先取消农业税。开展结对帮扶农村工作取得明显成效。行政村实现村村通水泥路、通公共汽车。农村“水改”成效显著，西部95％的自然村用上自来水。改造1389户农场职工危房，为646户低保无房户发放廉租房补贴，1.2万个家庭利用住房公积金贷款购买新房。2006年城镇居民人均可支配收入17676元，按可比口径年均增长6.4％，农渔民人均纯收入7006元，年均增长8.3％。

　　（六）政府自身建设取得新成绩，公共服务效能逐步提升。

　　民主法制建设加强。认真执行人大及其常委会的决议，自觉接受人大、政协和人民群众的监督，充分发挥各民主党派、工商联、无党派人士参政议政作用，三年共办理人大代表议案7件、建议563件，政协提案947件。政府法制工作成绩显著，编制建设法治政府五年规划，提请人大制定、修订地方性法规17件，建立政府法律顾问制度，“四五”普法工作受全国表彰。全面推行政务公开制度，加强行政监察和审计监督，查处一批违法违纪案件。

　　社会管理和公共服务职能加强。社会治安综合治理成效明显，刑事发案率连续三年下降，社会秩序保持良好。落实安全生产责任制，排查整治重大安全隐患，有效防范重特大事故发生。建立突发公共事件应急体系，成功处置了“东区一号”撞船等突发事件，应对突发事件能力增强。深化机关作风建设，创新公务员培训模式，加大培训力度，公务员综合素质有了新提高，公共服务意识和能力增强。国防建设和民兵预备役工作取得新成绩，双拥共建工作有新进展，第五次荣获全国“双拥模范城”称号。人口与计划生育工作连续三年获全省考核第一名。支持工会、共青团、妇联等人民团体开展工作，新闻出版、民族宗教、外事侨务、工商管理、对台、打私、人防、信访、档案、气象、仲裁、地方志、港澳流动渔民工作得到加强，老年人、残疾人和慈善事业都有新发展。

　　各位代表，回顾过去三年的发展历程，我们深感成绩来之不易！在此，我代表珠海市人民政府，向奋斗在各条战线的全市广大干部群众和特区建设者，向为珠海发展作出贡献的老领导、老同志，向各位人大代表、政协委员、各民主党派、工商联和社会各界人士，向中央和省驻珠单位，驻珠军警部队官兵，向所有关心支持珠海建设的港澳同胞、台湾同胞、海外侨胞和国际友人，表示崇高的敬意和衷心的感谢！

　　总结过去三年的工作，我们始终坚持在市委的领导下，紧紧依靠全市人民，形成团结和谐、心齐干事的工作局面；始终坚持走新型工业化道路，在壮大经济总量的基础上，注重提升经济质量和效益；始终坚持弘扬特区精神，把改革开放创新作为推动发展的根本动力；始终坚持可持续发展，努力实现经济发展和环境保护双赢；始终坚持把维护和发展人民群众利益作为工作的出发点和落脚点，努力让人民群众分享改革发展成果。所有这些，既是过去工作的宝贵经验，也是我市未来发展必须坚持的原则。

　　同时，我们清醒地认识到，珠海经济社会发展中还存在着一些突出问题，主要是：产业集聚不够充分，经济结构性矛盾突出，自主创新能力不强，经济质量和效益有待提高；重大基础设施配套还不够完善，“双港”辐射力不够强，完善立体交通网络任务艰巨；镇村经济比较薄弱，农民增收压力较大，被征地农民生活保障水平有待提高；特区意识有所淡化，改革和创新的锐气有所减弱，影响发展的体制机制性障碍仍然存在；公务员队伍建设有待加强，政府管理和服务水平仍需进一步提高。所有这些问题，我们必须高度重视，并要在今后的工作中采取有力措施加以解决。

　　二、今后五年的工作思路和奋斗目标

　　今后五年，是建设实践科学发展观先行示范市、构建和谐珠海的关键时期。政府工作的总体思路是：以邓小平理论和“三个代表”重要思想为指导，以科学发展观统揽全局，以改革开放和自主创新为动力，按照市第六次党代会的部署，发挥区位、环境、特区品牌三大优势，深入实施“工业强市”、“科教兴市”、“三产旺市”三大战略，坚持以人为本，速度与效益并重，发展经济与保护环境双赢，科学规划、合理布局，城乡区域协调发展五大原则，推动经济社会全面协调发展，构建和谐社会，加快建设珠三角现代化区域中心城市和实践科学发展观先行示范市。

　　今后五年，全市经济和社会发展的主要预期目标是：

　　──经济发展有新跨越。到2011年，全市本地生产总值翻一番，达到1500亿元，人均生产总值超过1万美元，工业总产值达到4500亿元，地方财政一般预算收入超过110亿元，固定资产投资超过500亿元，引进外资超过16亿美元，引进内资25亿元，进出口总额超过500亿美元，单位生产总值能耗降低15％以上，实现经济又好又快发展。

　　──自主创新能力有新提升。自主创新体系进一步完善，区域、产业、企业和产品竞争力有明显提高，形成一批创新型企业和产业集群，成为全省推进技术进步重点城市。科技投入明显增加，高新技术企业研发经费占销售收入的5％，全社会研究与开发支出占本地生产总值的2％。软件、生物医药产业年产值分别达到300亿元和100亿元，高新技术产品产值超过1800亿元，其中具有自主知识产权的高新技术产品产值所占的比重提高到30％以上，科技进步对全市经济贡献率达55％。

　　──城市建设有新突破。建成全覆盖的城市规划体系，城市基础设施更加完善，城市集散、服务和带动功能明显增强，华南沿海主枢纽港框架基本建成，珠三角现代化区域中心城市地位初步确立。新城区建设取得突破性进展。城市绿化覆盖率超过60％，城区居民基本用上管道燃气，城镇污水集中处理率达到80％，城镇生活垃圾无害化处理率100％，城市空气质量二级以上天数占全年的95％，生态环境建设继续保持全国领先水平。

　　──新农村建设有新成效。2011年，大部分镇的工业产值超百亿元，有条件的中心镇工业产值实现500亿元以上。农渔业总产值达50.5亿元，农渔民人均纯收入年均增长7％以上，力争达到1万元。农村富余劳动力实现5万人转移就业。农村“五保户”供养机制健全，农村最低生活保障实现应保尽保，农民养老保险覆盖率达到90％以上。完成农村特困户危房改造。生态文明村建设深入开展，农民生产生活条件进一步改善。

　　──和谐社会建设有新进展。创建“全国文明城市”，教育、文化、卫生、体育等各项事业全面发展。率先基本实现教育现代化，实现城乡一体化免费义务教育目标。公共卫生服务体系更加健全，确保每个镇有一个公立医院，全面完成121个农村卫生服务中心建设，人口计生事业进入全国先进行列。社会保障和社会救助体系更加完善。社会治安秩序保持良好，重大自然灾害、生产安全事故、重大环境污染、食品药品安全等突发事件预警处置体系全面建立，消防等应急设施全面加强，公共安全水平全面提高。

　　──人民生活水平有新提高。城镇居民人均可支配收入达23660元，年均增长6％。城镇登记失业率控制在3％以下，基本养老保险、基本医疗保险覆盖率达90％以上，新型农村合作医疗覆盖率达95％以上，千人拥有医生数2人、床位数4张。高中教育、高等教育毛入学率分别达到98％和43％以上，饮用水源水质达标率达到96％，城镇人均公共绿地面积超过14平方米，环境综合指数、居民主要健康指标、主要农副产品安全检测平均抽样达标率、药品安全抽样合格率等民生指标明显提高。

　　今后五年，要着力做好以下工作：

　　一是着力加快经济发展步伐，做大经济总量。坚持科学发展，大力实施“工业强市”、“科教兴市”、“三产旺市”战略，坚定走新型工业化道路，加快高新区、横琴经济合作区、高栏港经济区等经济功能区开发建设，积极发展高新技术产业，大力发展临港重化产业和先进制造业，重点加快发展旅游、物流等现代服务业，鼓励总部经济发展。提升外源型经济发展水平，增强国有经济竞争力，大力发展民营经济，壮大镇村经济实力，进一步转变经济增长方式，优化经济结构，提高经济发展质量与效益。

　　二是着力加快基础设施建设，增强城市集散和服务功能。全力推进以“一桥、两铁、双港”为重点的基础设施建设。推动港珠澳大桥尽早动工建设，积极推进广珠铁路、广珠城际轨道建设，建成高栏港区4个10万吨级集装箱码头、2个8万吨级公用石化码头、3个大宗散货码头、1个10万吨级液化天然气码头等港口设施，开发利用万山港区深水岸线资源。加快推进航空物流园区建设，充分发挥空港作用。加快西区、南区、海岛供水等工程建设，逐步推行城市分质供水，完善城市排水设施。规划建设通往西部地区的第二、第三通道，逐步将珠海大道等城市道路改造成快速干道，完善高速公路和快速干道配套路网建设。

　　三是着力推进自主创新，提升城市核心竞争力。以优化创新环境、强化企业创新主体地位、构建创新服务平台为重点，大力提升自主创新能力，建设创新型城市。全面落实自主创新各项政策法规，加大制度创新和体制创新力度，营造创新文化氛围。大力扶持自主创新龙头企业，培育自主创新企业群体。支持高校、科技园区和企业建立一批公共研发平台、公共测试平台、技术产权交易平台。发挥高新区人才、教育、科技、品牌等资源优势，把高新区打造成为我市自主创新示范区。培养和引进各类创新型人才，充分发挥企业家在自主创新中的核心地位。

　　四是着力推进改革开放，增创特区新优势。履行特区使命，弘扬特区精神，以特别之为，立特区之位，全面推进综合配套改革，努力在思想观念、发展模式、体制机制、对外开放、企业管理、城市建设与管理等方面实现新突破，逐步消除制约经济社会发展的体制性障碍。积极参与以粤港澳和泛珠三角为重点的区域合作，提高对外开放质量和水平，增强对全球资本、技术和人才的吸引力，继续发挥经济特区改革开放“试验田”和“窗口”作用，当好排头兵，建设实践科学发展观先行示范市。

　　五是着力实施“135”行动计划，建设高品位城市。高起点规划，高标准建设，高效能管理，使城市布局更合理，功能更完善，交通更便捷，环境更优美，努力实现“一年见美、三年变优、五年显特”目标，提升“海上云天，天下珠海”城市品牌。建立覆盖城乡的规划体系，切实提高城市规划、建设水平。规划建设新的市行政中心，加快推进横琴新城、唐家湾新城、金湾西湖城区和温泉新城、斗门中心城区等新城区建设。完善行政综合执法管理体制，提升城市信息化管理和公共服务水平。

　　六是着力实施“五项工程”，扎实推进新农村建设。大力推进强镇富民、安居乐业、生态文明、扶贫济困、固本强基五项工程。构建以工业化为主导、服务业为支撑、农业产业化为基础的镇村经济发展新格局。鼓励农民自主创业、自办企业，发展劳务经济和物业经济，促进农民收入持续增长。大力开展农村帮扶工作，全面发展农村社会事业。积极推进城市基础设施向农村延伸，城市公共服务向农村覆盖，城市现代文明向农村辐射，建设生产发展、生活宽裕、乡风文明、村容整洁、管理民主的社会主义新农村。

　　七是着力解决民生问题，努力构建和谐珠海。切实解决人民群众最关心、最直接、最现实的利益问题。落实就业扶持政策，增加就业岗位。健全社会保险、社会救助、社会福利和慈善事业相衔接的社会保障体系，扩大社会基本保险覆盖面，做实基本养老保险个人帐户。坚持教育优先发展，加强素质教育，办好大学园区，大力发展职业教育。深化医疗卫生体制改革，加强公共卫生、农村卫生和社区卫生建设。积极推动文化产业发展，构建公共文化服务体系。强化社会治安综合治理，完善矛盾纠纷排查调处机制，协调各方利益，维护社会公平正义。优化公共财政支出结构，逐年加大公共安全、社会福利、环境保护、科教文卫体事业的投入，使我市改革发展成果更好地惠及全市人民。

　　八是着力加强政府自身建设，提高依法行政水平。围绕建设法治政府、服务政府、责任政府和效能政府的目标，推进政府职能转变，在切实履行经济调节、市场监管职能的基础上，加强社会管理和公共服务职能。落实国务院《全面推进依法行政实施纲要》，严格按照法定权限和程序行使权力、履行职责。深化行政管理体制改革，创新公共服务体制，改进公共服务方式，提高行政效能。扩大政务公开，大力发展电子政务，方便群众办事和监督。加强公务员队伍建设，构建科学的考核评价体系，建立行政过错责任追究制度。加强廉政建设，厉行节约，降低行政成本。

　　三、2007年的工作安排

　　今年是新一届政府任期第一年，做好2007年的工作意义重大。我们要进一步增强紧迫感、责任感和使命感，充分调动一切积极因素，营造干事创业的良好氛围，抢抓机遇、乘势而上，围绕“科学发展、优化环境、改善民生、促进和谐、依法行政”的要求，做好市政府各项工作，推动全市经济又好又快发展，加快建设和谐社会，努力开创珠海改革开放和现代化建设新局面。

　　今年我市经济社会发展的主要预期目标是：本地生产总值增长13.5％，单位生产总值能耗下降3％，工业增加值增长16％，财政一般预算收入增长15％，社会消费品零售总额增长14％，固定资产投资增长14％，外贸出口增长12％，实际利用外商直接投资额增长10％，引进内资注册资本增长20％，城镇登记失业率控制在3％以下，城镇居民人均可支配收入增长6％，农渔民人均纯收入增长7％。

　　为实现上述目标，必须做好以下工作：

　　（一）推进新型工业化，增强产业实力。

　　实施“工业强市”战略。坚持大办工业、注重办大工业，把工业发展作为经济工作的主线，坚定走新型工业化道路，向工业要速度、要规模、要效益。市与区形成合力、各有侧重，市重点抓产业发展规划和重要基础设施配套，区重点抓园区建设和招商引资。全力培育和引进石化、民用飞机研发制造等带动效应大、竞争力强的大项目，实现PTA二期、华丰造纸、威尔加速器等项目建成投产，加快构建电子信息、家电电气、石油化工、电力能源、生物医药、精密机械制造六大工业产业基地，为实现工业跨越式发展打下坚实基础。

　　提高工业载体开发建设水平。载体不足和配套水平较低是当前制约工业发展最突出的问题。落实财政支持措施，积极拓展融资渠道，加快推进园区配套设施建设。按照“面积不变位置变、集中开发连成片”的思路，盘活村集体用地，清理排查园区存量和闲置用地。培育特色产业园区，加快产业集聚，促进园区集约发展，把单位建设用地的投资强度、GDP、税收和单位GDP能耗等作为考核园区发展的重要指标，不断提高园区综合开发水平。

　　加快内外源型经济发展。在巩固对港澳台地区引资基础上，加大对欧美日韩新等发达地区招商力度，大力吸引跨国企业集团前来投资，落实鼓励总部企业发展的政策措施，鼓励其设立地区总部、研发中心、采购中心，提高利用外资质量和水平。进一步优化投资环境，把引进内资作为招商引资工作新的着力点，大力引进国内大型企业集团、民营企业和上市公司，实现引进内资的新突破。大力实施“百优”民营企业培育工程，充分发挥促进民营经济发展联席会议制度的作用，努力创造平等竞争、一视同仁的发展环境，鼓励各种所有制经济共同发展。

　　强化为企业服务。构建市区镇三级联动、点面结合的企业服务体系，加强企业产前、产中、产后全程服务，全面提升市、区行政服务中心的服务质量和水平，全面推行项目代办制，加大督办工作力度，加强重点在建项目现场服务，以优良的政务环境改善产业发展环境。鼓励格力、伟创力等优势企业增资扩产、做大做强，在土地供给、基础设施配套、技改贴息、人才引进等方面给予优先支持。加强对中小企业服务，健全企业信用担保体系和社会化服务体系，培育中小企业成长壮大，营造“亲商、安商、富商”的良好发展环境。

　　（二）实施自主创新战略，推动经济增长方式转变。

　　推动企业成为自主创新主体。全面落实激励自主创新的配套政策，倡导和弘扬“尊重个性、鼓励探索、宽容失败、追求成功”的创新文化，优化创新环境。加快建立以企业为主体、市场为导向、产学研相结合的技术创新体系。加大科技投入，改革创新科技三项经费投放机制，提高财政资金使用绩效。引导和鼓励企业加大技术改造和技术创新力度。积极推动银企合作，推动创新型企业与资本市场对接，吸引风险资本扩大在珠海投资。

　　加强创新平台建设。修编高新区发展规划，注重产业发展与新城开发联动，努力把高新区打造成为我市自主创新示范区。重点建设一批科技企业孵化器，完善公共实验室及大型仪器共享、公共测试和技术产权交易等技术创新平台。办好大学园区，发挥大学园区智力优势和科技支撑作用，加快大学科技园建设，构建区域创新创业服务体系。

　　大力实施专利、品牌带动和人才战略。依法加强知识产权保护，严厉打击各种侵权行为。推动专利技术产业化，加强计量标准化建设。鼓励企业开发具有自主知识产权的品牌，政府采购适当向自主创新产品倾斜，奖励荣获国家、省名牌产品和商标的企业。完善人才政策，加快博士后流动站点建设，提高珠海对创新型人才的吸引力。高度重视发展职业技术教育，加快推进珠海城市职业技术学院新校区建设，大力培养技能型、实用型人才。

　　推进信息化建设。加强统筹规划，以信息技术应用为核心，加快旅游、交通、社保、社区服务、中小企业服务等重点领域信息化工程建设。提升有线数字电视网络功能和服务水平。加快推进企业信息化，支持亚仿公司在线仿真技术推广运用。积极推进农村信息化示范试点工作。

　　发展循环经济。将节能降耗目标责任纳入行政区、功能区绩效评价体系。尽快制定循环经济发展规划，启动试点工作，重点在现代农业、石化产业、能源产业率先探索发展循环经济。大力扶持节能产业发展，鼓励企业应用节能降耗技术，完成21家重点耗能企业节能降耗任务。发展再生资源产业，提高资源综合利用水平。加快城市天然气利用工程建设，推广使用清洁能源。

　　（三）发展现代服务业，调整优化经济结构。

　　重点加快旅游和物流业发展。充分发挥旅游业的龙头带动作用。发展温泉、海岛、游艇俱乐部等特色旅游产品，推进旅游产品转型升级。加快推进海泉湾度假城配套项目、东澳岛等重点旅游项目开发建设，办好国际航展、汽车赛等活动，加强旅游市场管理和区域旅游合作，努力把珠海建设成为粤港澳地区重要的游客集散地和主要的旅游目的地城市。编制物流业发展规划，依托机场、港口、高速公路等交通设施，利用保税区功能优势，规划建设物流园区，吸引国内外大型物流企业落户珠海。

　　促进房地产业健康有序发展。落实国家房地产调控政策，防止房价过快上涨。以满足群众基本住房需求为导向，加强住房建设规划，调整住房供应结构。合理调控土地供应规模和结构，优先保证中低价位、中小套型普通商品住房和廉租住房的土地供应。加快盘活烂尾楼，继续稳步推进城中旧村改造，提高房地产业开发建设水平。规范房地产市场秩序，加强市场交易和信息发布管理。

　　大力发展生产生活服务业。营造有利于服务业发展的良好环境，大力发展会议展览、信息咨询、会计和法律服务、经纪服务等生产性服务业。完善商业布局规划，提升拱北、吉大、新香洲商业区发展水平，加快西部地区大型商贸设施建设。支持果菜、肉类、水产品等专业批发市场发展。制定优惠政策，支持南方文化产业园建设，加快现代传媒、印刷出版、创意动漫、文化艺术服务等新兴服务业发展。

　　（四）深化改革扩大开放，增强发展动力。

　　深化市场经济体制改革。完善国有资产经营和监管体制。进一步发挥国有资本在基础设施、公用事业、现代物流、交通、能源等重点领域的引导和带动作用。发挥城资公司、汇畅公司、港口集团等国有资产资本运作平台作用，强化筹资融资、投资管理等功能。深化产权主体多元化改革，引入战略投资者参与国有企业重组，加快供水、公交等市政公用事业特许经营改革。发挥市公共资源交易中心作用，完善生产要素市场建设。继续整顿和规范市场经济秩序，严厉打击走私贩私、制假售假、偷逃骗税、非法传销、商业欺诈、商业贿赂等违法犯罪行为，加强反洗钱工作。支持行业协会等各类中介组织健康发展。

　　深化财税、金融和投融资体制改革。强化社会综合治税，提高税收征管水平。加强公共财政服务，完善部门综合预算改革，推行区级国库集中支付改革试点，实施政府收支分类改革和财政支出绩效评价，加强政府非税收入管理，深化政府采购制度改革。维护金融秩序，鼓励金融创新，积极推动地方中小金融机构改革重组。把握资本市场发展机遇，积极支持优质企业上市，扶持上市企业做大做强。逐步完善基础设施投资回报补偿机制，吸引外资和民间资本参与基础设施建设和运营。全面推行政府投资项目代建制，提高项目建设管理水平。

　　以横琴开发为契机推动区域合作和对外开放。按照“科学规划、明确定位、积极协调、主动开发”的思路，抓紧完成横琴控制性详细规划，加快重点项目审核报批工作，上半年要落实一批项目动工建设，实现横琴岛开发有新突破。重点推进珠海与港澳在货物贸易、服务贸易和投资贸易等领域的合作，创造条件建立香港服务贸易业转移基地。推进珠港机场全面合作，不断拓宽合作领域。发挥珠澳跨境工业区特殊的区位和政策优势，在产业开发的同时，探索新的监管模式。加强对台经济技术合作与交流。

　　提高口岸通关便利化水平。以提升口岸城市功能为目标，编制口岸发展规划，优化布局，合理划分功能，加强协调服务，不断完善口岸配套，推进“大通关”建设。推广“一站式”电子验放系统服务，建设电子口岸，提高通关效率。依托口岸优势，大力发展口岸经济。

　　（五）全面启动实施“135”行动计划，优化城市环境。

　　全力抓好重大基础设施项目建设。珠海港、广珠铁路、港珠澳大桥等重大基础设施项目关系珠海未来发展，必须全力以赴，加快推进。启动珠海大道改造和井岸二桥建设工程，修通金唐路，新建凤凰山隧道、金凤路、翠屏路、三台石路北段、南横路洪屏段、省道272线。加快高栏港区两个5万吨级集装箱码头建设、加紧做好4个深水集装箱码头和大宗干散货码头的前期工作。重视电力网规划建设，抓紧220千伏输变电工程建设，做好500千伏输变电工程前期工作。加强水资源管理，加快竹银水源系统等供水设施建设，进一步提高城市供水安全保障能力。加快城乡水利防灾减灾工程和河道畅通工程建设，提高防洪排涝能力。

　　提高规划和土地管理水平。完成《珠海城市空间发展战略研究》等总体规划、唐家湾新城等分区规划、主城区及各新城近期建设地区的控制性详细规划，开展主要城市通道等28项城市设计。高水平开展金湾西湖城区、温泉新城和斗门中心区的规划建设，加快西部地区城市化进程。建立健全规划咨询、论证、公示、听证制度，严格执行规划。认真执行国家土地宏观调控政策, 全面实施土地出让“招拍挂”制度。加大土地收购储备力度。抓紧完成市区镇土地利用总体规划修编，科学编制年度土地供应计划，优先保障重大建设项目、工业园区和新城区建设用地。深化征地制度改革，严格保护耕地。

　　实现“一年见美”目标。加快实施与市民生活息息相关的城市建设和改造项目。改造、美化一批城市道路，建设林荫大道，新建和改造一批公园和休闲广场，改造老旧住宅小区街巷。完善物业管理配套法规，提升物业管理水平。建设数字化城市管理系统，加强城市管理综合执法，规范招牌、广告设置，进一步净化和美化城市环境。

　　加大环境保护力度。实施生态市建设规划。落实主要污染物总量控制责任制，抓好“十项环境重点工程”，继续完善主城区排污系统管网，新建三灶、井岸和高栏港等三座污水处理厂及其配套管网工程。加快前山河和市区排洪渠综合治理。建设西坑尾垃圾处理厂、医疗垃圾焚烧处理设施，新建西区垃圾处理场，对已封场的垃圾填埋场进行生态恢复。建设市环境监控应急指挥中心，新建和完善一批大气、水质、污染源等环境监测设施。依法严格控制开山取土，严禁破坏山体和植被，保护绿水青山。

　　（六）促进镇村经济发展，推进社会主义新农村建设。

　　提升农村公共服务水平。加快推进社会主义新农村“五项工程”建设，促进城乡协调发展。推动社会公共资源向农村倾斜，扎实推进农村对口帮扶工作，增加财政资金对农村就业服务、社会保障、生态保护、社会治安和教育、卫生、文化事业等方面的投入。市区镇共同努力，完善村镇道路支线、辅道等配套设施建设，使城市道路、供水、排污、供电、通信等基础设施向镇村延伸。

　　加快发展农村经济。以工业园区和中心镇为依托，发展一批工业强镇。鼓励村集体和村民在工业园区周边发展第三产业，引导农村劳动力向非农产业转移。实施农村集体建设用地使用权流转制度，积极推进工业向园区集中、农村人口向城镇集中、农业用地向农业龙头企业和种养大户集中。以抓工业理念抓好农业，以良好基础设施保障农业，以先进科技提升农业，以现代农业制度推动农业，发挥农业龙头企业的带动作用和农村合作经济组织的纽带作用，提高农业产业化、集约化水平。建设生态农业科技园，培育名优特农产品，扶持种子种苗基地、农产品加工流通企业发展。加大农民技术培训力度。建立补偿机制，支持农田保护区和水源保护地区经济发展。

　　开展环境优美乡镇和生态文明示范村建设试点。把镇、村建设纳入全市统一规划。今年完成20个社会主义新农村试点村（镇）建设规划工作，注重保护乡村传统特色。整合资源，稳步推进中心村建设和旧村改造。搞好农村街院净化、街道亮化、村庄绿化、环境美化，建设一批生态文明示范村，改善农村生态环境和人居环境。落实村民自治，完善村务公开、财务公开制度。开展争创“五好文明家庭”、“文明农户”活动，倡导文明新风，建设和谐乡村。

　　发展海洋经济。编制海洋功能分区区划，加大海洋综合开发与资源保护力度。深化万山深水港规划，推动黄茅岛液化天然气项目和桂山油库改造，发展海岛中转仓储业。发展海岛旅游业。抓好人工鱼礁建设和管理，增殖渔业资源。加快桂山岛供水工程建设，推行海岛垃圾集中处理，改善海岛生产生活条件。

　　（七）致力改善民生，推进和谐珠海建设。

　　高度重视解决群众切身利益问题。千方百计实施“教育优先”战略。加大教育投入，巩固“教育强市”成果，优化教育资源配置，提高西部地区农村和海岛学校办学质量，增加优质学位，努力满足人民群众对优质教育的需求。从今年秋季起免收全市农村义务教育阶段学生书本费。千方百计提高群众健康保障水平。加强农村三级医疗预防保健网络建设，今年新建100个农村卫生服务中心，基本解决农民“看病难”问题。完善困难群体医疗救助制度，制定免费婚检和孕检实施办法，打击医药购销领域商业贿赂，努力解决群众“看病贵”问题。千方百计保障公交优先发展。加大城市公共交通投入，调整优化公交线网布局，加快轨道交通、综合换乘枢纽的规划建设，合理安排站场和配套设施建设，完善低票价的补贴机制，倡导文明交通，既确保广大群众出行安全、便捷，又缓解交通拥堵压力。千方百计保障食品安全。实施食品放心工程，支持绿色、有机农产品生产基地和副食品专区建设，严格执行市场准入制度，建立长效管理机制，确保人民群众饮食安全。千方百计提高就业保障水平。加快健全覆盖城乡的公共就业服务体系，大力实施再就业援助工程。规划建设“三位一体”公共实训基地，提高就业培训质量。多渠道开发就业岗位，资助500名贫困家庭和“零就业家庭”子女免费就读我市中高等职业学校，并推荐就业。千方百计提高群众对城市生活环境的满意度。探索堵疏结合、标本兼治的城市管理新路子，大力治理城市乱摆卖、乱停放、乱搭建现象，整治城市“卫生死角”，加大对汽车尾气排放、噪声污染等方面的执法力度。引导农贸市场规范经营，改善农贸市场购物环境。千方百计解决困难群众生产生活问题。加快红旗、平沙和白藤农场体制改革，推动农场行政融入地方，管理融入社会，经济融入市场。今年市安排5000万元用于支持农场危房改造，每户补贴资金从1万元增加到2万元。发挥红十字会和慈善组织扶贫济困作用，大力实施助学、助残和单亲特困母亲扶助工程，确保困难群众得到及时救助。

　　完善社会保障体系。扩大农村养老保险、未成年人医疗保险和农民工参加工伤保险的覆盖率。积极探索构建“大病救助、中病保险、小病免费”的医疗保障体系。今年市安排720万元补贴资金，将新型农村合作医疗筹资标准由每人每年60元提高到80元，其中市财政每人补助30元，参保农民医疗费报销最高可达1.5万元。合理提高社保费在征地补偿费中的比重。全面落实最低生活保障制度，推进分类施保工作，实现应保尽保。完善社会救助体系，支持社会福利事业发展，建设市福利院。改扩建市计生服务中心，加强流动人口计生管理和服务。保障妇女儿童权益，发展养老服务业和残疾人事业。

　　建设“平安珠海”。坚持严打、严防、严管、严治并重，加强社会治安综合治理，做优做强珠海治安品牌。落实科技强警，加快建设城市治安视频监控、城市火灾远程监控、车辆号牌自动识别系统，重点加强市区老旧小区安全设施建设，加强出租屋管理，强化路面巡控，保持对刑事犯罪高压打击态势，增强人民群众安全感。加大投入，争取用三年时间基本消除消防基础设施投入欠账，积极发展多种形式的消防队伍。以铁的手腕抓安全生产，落实政府安全生产监管责任制和企业安全生产主体责任，加强安全生产监管队伍建设，对危险化学品、道路和水上交通、建筑施工、森林防火、消防安全开展专项整治。全面推广建筑工人“平安卡”管理制度，加强职业安全卫生监管。加大应急设施建设投入，成立市、区应急管理指挥中心，提高突发公共事件应急处置和救援水平。建设“海上110”，维护海上安全。高度重视信访和人民调解工作，引导群众合理合法表达利益诉求。认真解决土地征用、城镇拆迁、单位改制中涉及群众切身利益的问题，维护社会稳定。抓好“五五普法”工作，大力嘉奖见义勇为行为，加强法律援助，引导和鼓励企业承担社会责任，保障外来务工人员合法权益。

　　全面启动创建全国文明城市工作。深入开展“八荣八耻”社会主义荣辱观教育，提高公民思想道德素质。发展壮大文化事业和文化产业，保护文物和非物质文化遗产，加快古元美术馆、市档案馆、市博物馆和特色文化站改扩建工程。加强公众体育设施建设，发展公益性社区健身服务。加强国防建设和民兵预备役工作，加强双拥共建工作，做好现役军人家属、伤残军人、军烈属的优抚工作，争创第六次“全国双拥模范城”。继续做好民族、宗教、对台、外事、侨务、统计、人防、档案、仲裁、气象、地方志和港澳流动渔民工作，支持和保障各人民团体履行职能，共同推进和谐社会建设。

　　（八）加强政风建设，提高为民服务水平。

　　全面履行政府职能。推进行政体制改革，合理划分市、区、镇的权责，逐步建立权责对等的运行机制。牢固树立“全市一盘棋”的观念，抓好市委、市政府各项决策和工作部署的落实。全面推行政府部门目标管理，落实部门主办责任制和以行政首长为重点的行政问责制，奖优、治庸、罚劣。公务员要有作为，但不能乱作为，要把心思凝聚到珠海发展上、把精力集中在工作落实上，切实履行职责，确保权力不被滥用，提高政府的执行力和公信力。

　　积极推进依法行政。认真贯彻行政许可法，完善行政复议、行政赔偿和政府法律顾问制度。坚持重大事项集体决策、专家咨询、社会公示与听证等制度，认真听取民主党派、工商联、无党派人士和各人民团体的意见，广开市民建言献策渠道，提高依法决策、民主决策、科学决策水平。认真贯彻执行人大及其常委会决议，坚持向人大报告工作，及时向政协通报情况，自觉接受监督。办好人大议案、代表建议和政协提案。

　　树立服务型政府形象。各级政府公务员来自于人民群众，为群众服务是本身的职责。党组织和人民群众选择了我们在公务员的岗位上，我们就要奋力拼搏，全心全意为人民服务，把自己的智慧和力量奉献给珠海的事业，以实际行动，对珠海人民负责，对珠海发展负责。人民群众对政府提高服务水平期望很高，今年要着重解决“办事难”问题。各级政府机关要始终做到“平事快办，急事特办，大事集体办，难事千方百计办”，为企业、为市民提供优质高效服务。大力实施“阳光工程”，推进政府系统廉政建设和反腐败工作，强化行政监察和审计监督，坚决纠正损害群众利益的不正之风，树立“为民、务实、清廉”的政府形象。

　　各位代表！珠海的发展蓝图已经绘就，今后五年市政府工作责任重大，任务光荣而又艰巨。让我们紧密团结在以胡锦涛同志为总书记的党中央周围，高举邓小平理论和“三个代表”重要思想伟大旗帜，全面落实科学发展观，在党中央、国务院、省委、省政府和市委的坚强领导下，团结带领全市人民锐意进取，扎实工作，加快建设实践科学发展观先行示范市步伐，为把珠海建设成为“最适宜居住、最适宜创业、最富有魅力”的现代化区域中心城市而努力奋斗！
